# Short-term aerobic exercise for depression in acute geriatric psychiatry: study protocol for a randomized controlled trial

**DOI:** 10.1186/s13063-022-06567-4

**Published:** 2022-07-30

**Authors:** Laura Elani Schulte, Tim Fleiner, Rieke Trumpf, Daria Wirtz, Thiemo Schnorr, Wiebren Zijlstra, Peter Haussermann

**Affiliations:** 1 Institute of Movement and Sport Gerontology, German Sport University Cologne, Cologne, Germany; 2 Department of Geriatric Psychiatry & Psychotherapy, LVR Hospital Cologne, Wilhelm-Griesinger Straße 23, 51109 Cologne, Germany

**Keywords:** Old-age depression, RCT, Endurance training, Intervention, Hospital-setting

## Abstract

**Background:**

Major depression is one of the main mental illnesses in old age, with acute exacerbated episodes requiring treatment in geriatric psychiatry. A meta-analysis showed that aerobic exercise in moderate intensity has large effects in older adults with major depression, but there is no evidence of aerobic exercise in geriatric psychiatry. Therefore, this study aims to analyze the feasibility and effects of an ergometer-based aerobic exercise on depressive symptoms.

**Methods:**

A single-center randomized controlled trial will be conducted in an acute geriatric psychiatric hospital. Inpatients allocated to the intervention group will receive a 2-week aerobic ergometer program. The control group will receive seated flexibility exercise in addition to usual care. The overall effects on the patients’ depressive symptoms will be measured by clinical global impression of change (CGI) as the primary outcome. Changes in depressive symptom domains, physical (in)activity, and aerobic performance as well as the dosage of applied antidepressants will be examined as secondary outcomes.

**Discussion:**

This short-term aerobic exercise program is expected to decrease depressive symptoms in acute exacerbated periods in older adults. The results may increase the evidence for implementing physical activity interventions in acute hospital settings. The disease-related motivation for exercise in acute exacerbated depressive periods will be the most challenging aspect. The treatment of depression requires new cost-effective approaches, especially in acute geriatric psychiatry with potential benefits for patients, family members, and clinicians.

**Trial registration:**

German Clinical Trial Register ID: DRKS00026117

**Trial status:**

Protocol Version 1.2 dated February 23, 2022. By February 23, 2022, the trial had recruited a total of 15 participants in two wards at the Department of Geriatric Psychiatry at the LVR-Hospital Cologne. Recruitment started on November 12, 2021. The recruitment is expected to continue for at least 12 months.

## Background

Depression is a common disorder in old age [1] that affects around 7% of the older global population [2]. In Germany, 1.4 million adults above 65 years suffer from clinical depression, representing 8.1% of the population with 8100 cases per 100,000 residents [3]. The Global Burden of Disease (GBD) Study by the World Health Organization indicated that depression is one of the leading causes of disability [4]. The pathophysiology of depression has not yet been clarified, but the most common theory is the “monoaminergic hypothesis,” which identified a change in serotonin, dopamine, and/or norepinephrine metabolism [5, 6]. Neuroinflammation and chronic stress due to disruption of the brain-derived neurotrophic factor (BDNF) or transforming growth factor-β1 are also discussed in the context of the disease with negative effects on neuroplasticity [7].

Late life depression is associated with a higher risk of suicide [8] and decreased functioning [9], which comes with limitations in activities of daily living (ADL) [10] and leads to a deterioration in quality of life [11]. Besides the main affective symptoms like low mood, loss of interest, and fatigue [12], depression is a major predictor for cardiovascular and neurodegenerative diseases and increases mortality risk in older adults by 28% [13].

Acute exacerbated periods of the disease are treated in acute geriatric psychiatry, with an average hospital stay of 4 weeks and respective outpatient aftercare [14]. Antidepressant drug therapy is considered as first-line treatment with selective serotonin re-uptake inhibitors [15]. A recent review recommends physical activity as a complementary treatment approach to antidepressant drug therapy [16]. The authors concluded that older patients with late-life depression, who are at high risk of developing dementia, could benefit from this synergistic effect in particular. The biologically-based impact of physical activity on neuroplasticity, such as hippocampal neurogenesis, has already been explained [17]. Aerobic exercise has an impact on tryptophan hydroxylase, which is needed for the synthesis of serotonin [18] and increases BDNF levels [19–21], thus stimulating neuroplasticity and neurogenesis [16].

A short 10-day inpatient exercise treatment of major depressive episodes in middle-aged adults, aged 49 ± 13 [22] and 45.3 ± 10.6 [23] showed large effects favoring the aerobic intervention [22, 23] over stretching or no intervention. The aerobic exercise included brisk walks or jogging [23] or treadmill walking [22]. Meta-analyses showed that physical exercise is associated with significantly lower depression symptoms in older adults [24, 25]. Physical activity has the potential to positively influence mood in depressive disorders. There are several mechanisms of action for this, such as hormonal changes, effects on neurogenesis, inflammation, and oxidative stress, to changes in corticular activity and structure [25]. Studies included in the meta-analysis were conducted in an outpatient setting or in nursing homes with a minimum treatment period of 3 months. In particular, aerobic exercise and moderate intensity showed large effects in the treatment of depression [25]. A recent meta-analysis reported that aerobic exercise, such as walking or ergometer-based cycling, can be considered as safe and natural training for the older population [26].

To date, there is no evidence of short-term aerobic exercise in the treatment of old-age depression. Considering the positive effect on short-term inpatient treatment for a duration of 10 days in middle-age adults [22] and the lack of evidence in acute geriatric psychiatry, we aim to set up a randomized controlled trial (RCT) investigating the effect of aerobic exercise in older adults during acute exacerbated periods of major depression.

## Objectives

The primary objective of this trial is to analyze the feasibility and effects on depressive symptoms within a 2-week aerobic exercise program for inpatients in acute geriatric psychiatry.

We hypothesize that the intervention group (IG), carrying out a 2-week-aerobic exercise program in addition to treatment as usual (TAU), shows effects on depressive symptoms and physical activity at post-intervention as compared to the control group (CG).

## Methods

### Study design

**Fig. 1:**
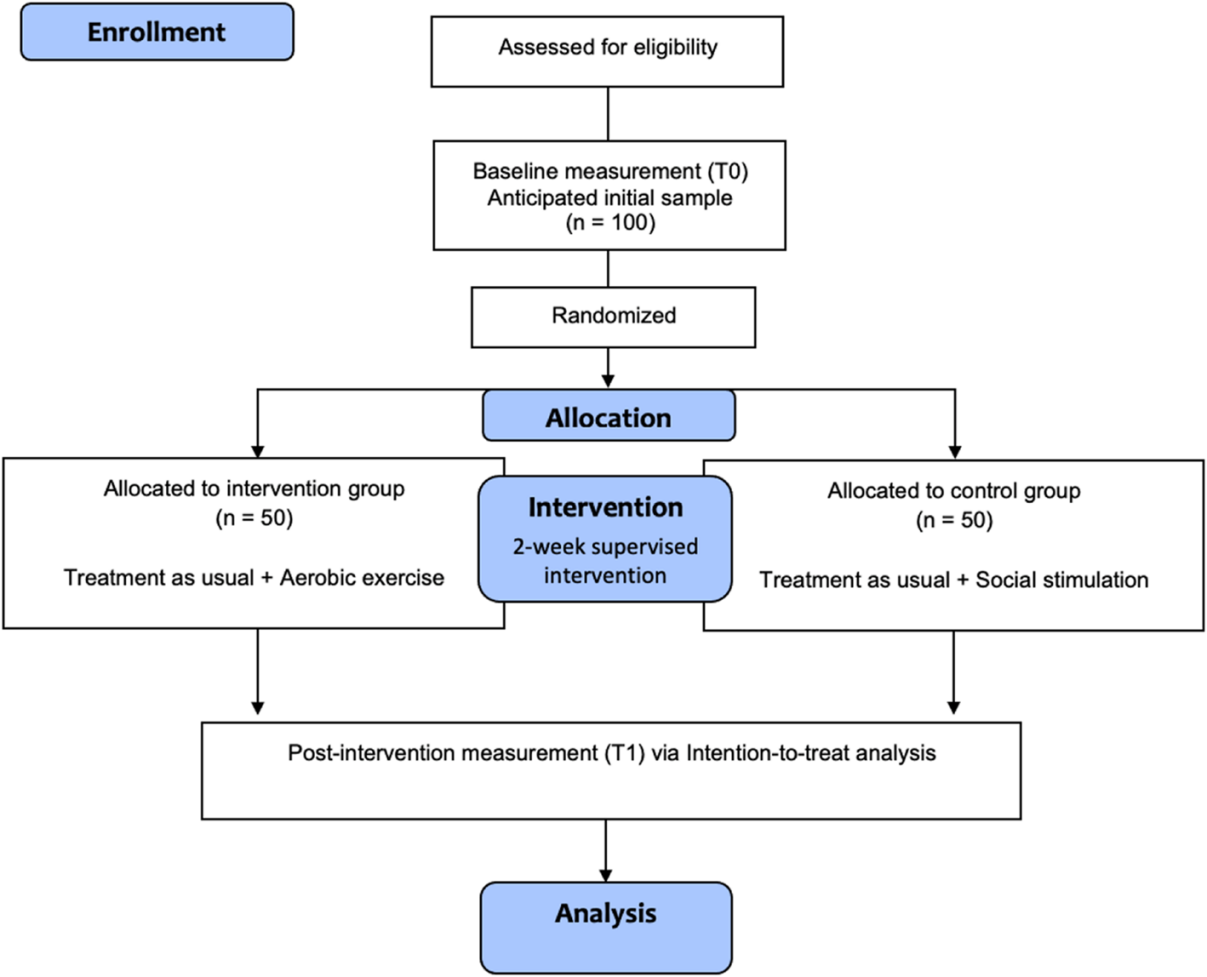
Study flow-chart

A monocentric randomized controlled superiority trial with pre- and post-intervention assessment will be conducted with 1:1 allocation ratio in the department of Geriatric Psychiatry at the LVR-Hospital Cologne (Fig. 1). The Ethics Commission of North-Rhine Medical Chamber obtained ethical approval (reference number: AZ 2018192). This trial is registered in the German National Register of Clinical Trials DRKS00026117. The protocol is reported according to the Spirit guidelines.

### Sample

Patients from two hospital wards will be screened for eligibility by a senior psychiatrist who is not involved in the study team. The inclusion and exclusion criteria are presented in Table 1. Written informed consent from the patient’s legal guardian or the patient was required. In accordance with the ethical approval and the task force on ethical and legal questions of the Association of Neuropsychopharmacology and Pharmacopsychiatry, the following consent for participation will be conducted [27]: With a score < 20 on the Mini-Mental Status Examination (MMSE) [27], the patient’s legal guardian was asked to give informed consent; for scores of ≥ 20 and the ability to explain the content and aims of the project in the patient’s own words, the patient was considered to be able to give the informed consent on its own.

**Table 1:** Inclusion and exclusion criteria

Inclusion criteria	Exclusion criteria
Diagnosis of depression according to ICD-10 (F31.3–F34)	Clinical exclusion of delirium (based on CAM); combined diagnosis of depression and an alcohol-induced neurodegenerative disease
Minimum length of stay of 3 days before enrollment in the study	Acute and/or severe cardiac disorder, neurological disease, and/or chronic orthopedic disorder
MMSE ≥ 19 points and the ability to explain the content of the study in the patients’ own words^a^	MMSE < 19 points
Prescription for exercise therapy (this includes cardiovascular capacity)	

*ICD-10* International Statistical Classification of Diseases and Related Health Problems, *CAM* confusion assessment method [28], *MMSE* Mini-Mental Status Examination, maximum score of 30 points, higher score indicating better cognitive functioning

^a^This approach is consistent with the Society for Neuropsychopharmacology and Pharmacopsychiatry Working Group on Ethical and Legal Issues [27]

As part of routine care, baseline characteristics are recorded within the geriatric assessment, which is carried out by a nursing and medical staff member of the LVR-Hospital Cologne. The geriatric assessment includes sex, age, weight and height, clinical diagnosis, MMSE [29], and Timed Up and Go Test (TUG) [30].

### Sample size calculation

The required calculation of the sample size is performed by using G*Power 3.1 [31]. Based on the results of the current state of studies [24] a moderate effect size is assumed for the change in depressive symptoms. The effects of Bridle’s pooled analysis show an effect size of -0.44 SMD for studies with an active control group. This corresponds most closely to the present methodology with an active control group. The sample required to detect statistically significant differences in the depressive symptoms for a two-tailed test of the proportions with an effect size of d=0.5 was calculated for the primary objective. Based on this calculation, we will include 100 patients in the trial, with 50 allocated to each study group. The allocation rate is 1:1, two measurements, non-parametric analysis due to ordinal characteristics of the instruments and a-error of 0.05 and a power of 0.95 (1-ß-error) according to Cohen [32].

### Allocation

With an informed consent and pre-assessment, randomization will be performed via stratified randomization. An organizational staff member, who is not part of the study team, will perform the group allocation. Study randomization was done through Study Randomizer (2017), a web-based randomization service [33]. In order to achieve the best possible balance between the two study groups, the factors, sex, age, and MMSE score [29] will be weighted 1:1 in the program. Patients are sequentially numbered, no conclusion can be drawn about the patients, with the exception of the study director who takes the patient's informed consent.

### Outcome measures

All outcomes will be measured by trained and experienced assessors from nursing and medical staff members, blinded to group allocation. With written consent to participate, patients were assigned an identification number (ID). The consent forms are kept in a locked cabinet for 10 years. All other data is stored via the hospital information system.

In cases of accident or injury, concerns of the medical staff members or unwillingness of patients to participate during the interventions, unblinding is permissible. For participants who suffer unexpected harm from trial participation receive medical care. The intervention will be modified immediately in response to harms or worsening patient’s conditions. If no improvement is achieved, the patient will be excluded from the study. Other criteria of discontinuing are, concerns of medical team members or if the patient withdraws his participation in the intervention. Adverse events related to the intervention are documented in detail.

Primary and additional outcome variables are presented in Table 2. The time points for the primary outcome measures are a maximum of 1 week pre- and post-intervention. The adherence to intervention and the documentation of adverse events will be recorded to investigate the feasibility of the exercise intervention. A potential relation of adverse events to the intervention will be evaluated by a senior old age psychiatrist, who is not part of the study team.

**Table 2:** Objectives and instrumental set-up of the trial

	Variable	Instrument
**Primary outcome**	Depressive symptoms	CGI – Global improvement scale [34]
**Secondary outcome**	Depressive symptoms	BDI II [35] HAMD [36]
	Physical (in)activity	Body fixed Motion Sensor (McRoberts, The Hague, NL)
	Endurance performance	6-min walk test [37]
	Application of antidepressive medication	Fluoxetin equivalents (FED) [38]

### Primary outcome

The primary outcome of the intervention is the change in depressive symptoms rated by the clinical global impression of change (CGI). The CGI is performed by the clinician and includes a 1- to 7-point rating scale of the global improvement due entirely to treatment [34].

### Secondary outcomes

As a secondary outcome, the symptom domains and the severity of depressive symptoms will be measured by self- and peer-rated assessments. The Beck Depression Inventory (BDI II) [35] is a self-rated scale which is conducted with the patient in interview form. The Hamilton Rating Scale for Depression (HAMD) [36] is performed by the nursing staff members. Mobility-related measurements, such as physical in(activity), will be performed with the Dynaport Move Monitor + (MM+; McRoberts, The Hague, NL). The MM+ consists of an accelerometer, a gyroscope, a magnetometer, a barometer and a temperature sensor. The raw data will be processed using the manufacturer’s own algorithms. The corresponding output includes the type of activity and body positions (walking, stair walking, cycling, shuffling, standing, sitting, and lying) and an additional category not-worn, corresponding MET-values, activity duration, and number of steps per 60-s epoch. Data will be collected for a 48-h period, starting and ending at 12 am on days one and three. The sensor is attached to the lower back approximately 3 cm to the right of the fifth vertebra of the lumbar spine (L5) with a waterproof self-adhesive foil (Opsite Flexifix, Smith and Nephew, London, UK) [39]. The participants will be asked not to remove the sensor during the measurement period.

To assess the endurance performance, the 6-min walk test [37] will be performed at pre- and post-assessment. The patients will be asked to walk a maximum distance in 6 min. The walking speed can be determined by the patient himself and changes and breaks will be allowed.

The applied total dose of antidepressant medication will be assessed by converting the fluoxetin equivalent doses (FED) and the medication on demand in mg/day according to the dose equivalents calculation of Hayasaka et al. [38].

**Fig. 2:**
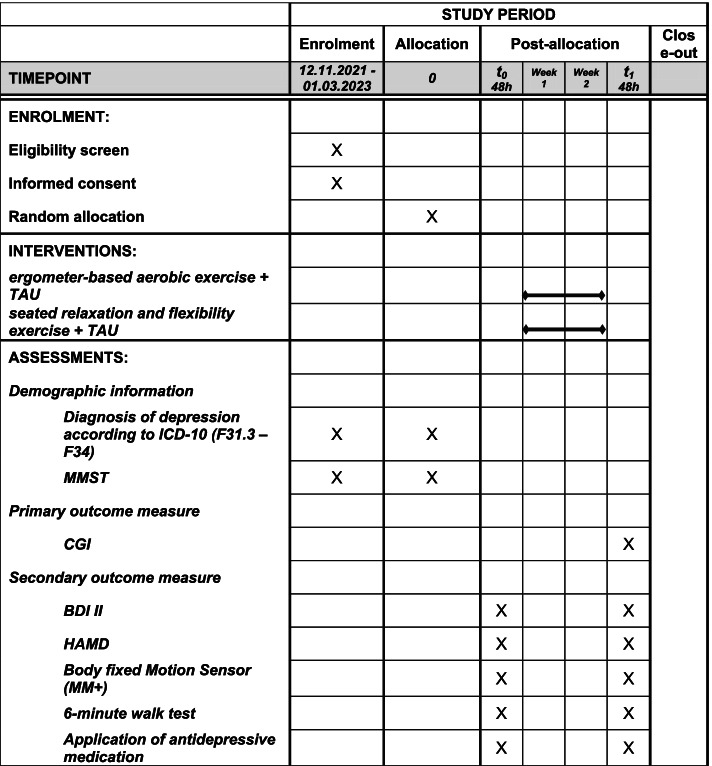
SPIRIT figure. TAU, treatment as usual; ICD-10, International Statistical Classification of Diseases and Related Health Problems; MMSE, Mini-Mental Status Examination; CGI, clinical global impression scale; BDI II, Beck Depression Inventory; HAMD, Hamilton Rating Scale for Depression; MM+, Dynaport Move Monitor +

The phases of the trial and the data collection at each timepoints are presented in Fig. 2.

### Intervention

#### Intervention group

Participants allocated to the IG will receive a 2-week ergometer-based aerobic exercise three times per week on non-consecutive days and a duration of 20 min, twice per day. The training can be completed using four different modalities, seated-ergometer bicycles for the upper or lower limb (Emotion Fitness, Inc., USA), rowing ergometer (Concept 2, Inc., USA), or ski ergometer (Perform Better Inc., USA) placed on the hospital wards. This approach is planned to provide low-threshold access and variety in exercise selection, allowing participants to choose a preferred style of moving at each session to complete the weekly requirements. If the patient refuses or is hindered for any reason, the patient is asked to participate once again on the same day. The adherence to the intervention will be recorded by study reports and the training intensity will be monitored by the heart frequency (Polar A300, Polar Electro Oy, Finland).

The training will be performed individually and under constant supervision by research staff. Patients will be instructed on the adequate use of exercise machines before and during each training session. The participant will receive a fitness and activity tracker with a heart rate (HR) monitor (Polar UNITE, Polar Electro Oy, Finland) and be asked to rate the perceived exertion (RPE) with the 6-20 Borg Scale [40] after 10 min (half time of intervention) and after 20 min (end of intervention). The respective training HR will be determined using the formula 220 minus the patient’s age [41]. Each session will begin with a 2 min warm-up of ergometer use with a HR under 50% of the estimated peak heart rate. After 3 min, the participant will be asked to increase the intensity up to 55% of the estimated peak heart rate. After 18 min, the participants will be instructed to decrease the intensity to their own wish to initiate the cool-down. The research staff will collect the exercise data from each session, including the total time of training, average HR and Borg Scores.

#### Control group

Patients allocated to the CG receive a seated relaxation and flexibility exercise with the same level of social interaction as compared to the IG in addition to TAU, which is not expected to show changes [42]. This control intervention is assumed to be an appropriate exercise placebo intervention with very low-intensity exercise, requiring only minimal muscular strength and aerobic capacity. Throughout the study, all participants will receive TAU, such as antidepressants or physiotherapy. It will also include exercise therapy for 45 min twice a week as part of the routine care.

### Analysis

The trial will be analyzed by using the intention-to-treat principles, in which all patients with baseline data are included in the analysis. Adherence to intervention will be collected descriptive at each training session. The data are stored on the clinic’s own servers, which comply with a heightened security system. Missing data that are not directly related to adherence will be imputed, in order to include all participants. Differences between IG and CG from baseline to post-intervention will be determined by one-way repeated measure ANOVA for parametric and normally distributed data or the Friedman one-way repeated measure analysis of variance for non-parametric or non-normally distributed data. The level of significance is set at alpha ≤ 0.05. All computations will be performed using SPSS software (IBM Corporations, Armonk, NY, USA). No committee for data monitoring and auditing is planned.

## Discussion

The primary aim of this study is to investigate the effect of aerobic exercise in older adults with major depression in acute geriatric psychiatry. Meta-analyses reported only a few adverse events in older adults during ergometer-based aerobic training [26]. We expect a corresponding adherence to the training intervention, due to exercise sessions conducted on the wards and multiple exercise sessions within an exercise day. Based on the previous research of positive effects in middle-aged adults with clinical depression [22] and the positive outcomes of aerobic exercise with older adults [25], we assume the feasibility and a significant change in depressive symptoms, due to a number of mechanisms of action, such as hormonal changes, effects on neurogenesis, inflammation and oxidative stress [25].

This single-center randomized controlled trial will be the first, to the best of our knowledge, to evaluate a short-term aerobic exercise on different ergometers in a hospital-based setting with older adults suffering from clinical depression.

The short duration of 2 weeks is derived from the median length of stay in the hospital of 32 days in older inpatients with depression [14]. The intervention takes place on the wards of a geriatric psychiatric unit and will begin no earlier than 3 days after admission to allow patients to acclimatize. This schedule seems feasible to include most patients and preserve a low dropout rate due to early discharges.

A serious issue in the hospital setting is the high level of physical inactivity among inpatients [43, 44]. A recent umbrella review indicates that higher physical activity levels in older adults have a 21% reduction in incident depression compared to older adults, who have lower levels of physical activity [45]. Physical inactivity leads to other risk factors such as sarcopenia [46, 47], loss of independence [48], and disability in ADL [49, 50]. Recent recommendations during hospitalization suggest that older inpatients should be as active as possible and incrementally enhance movement in everyday activities [51]. In addition, it is recommended that the influence of the physical environment should also be considered to enable older people to be active with portable adaptations and equipment for indoors and outdoors [51]. For this study, new types of ergometers will be placed on the wards to facilitate an environment that promotes exercise. From our point of view, this could counteract the loss of interest and decreased impulse known as key aspects in exacerbated periods of major depression. From our clinical experience, the variety of ergometers should increase the patients’ willingness for participation. It also offers advantages, for example when patients experience fatigue or pain in the lower limbs, they can still exercise while sitting on the ski ergometer. Therefore, this type of study is an important opportunity to strengthen physical activity in standard treatment.

This will be the first single-blinded RCT to examine physical interventions for the treatment of depression in an acute hospital setting. The outcomes of this trial can be directly transferred and implemented in the hospital. In order to include as many acute geriatric patients as possible, the inclusion criteria were chosen to include patients with mild cognitive impairment and bipolar disorder with depressive episodes.

Some limitations must also be considered; diverse factors in the TAU process could influence the success of decreased depression symptoms. The TAU, especially the pharmacological therapy, is expected to be effective in the treatment of depression symptoms. Another limitation is that the severity of depression symptoms, for example, a minimum score of the HAMD, is not an inclusion criterion. This study will be conducted in a geriatric psychiatry unit, where all of the included patients have a clinical diagnosis of depression, so symptom severity can be assumed.

The results of this study may help to increase the evidence in implementing and investigating physical activity interventions in acute hospital settings. During this stay, the patient receives an initial input of ergometer-based physical activity intervention on the ward, which is offered again in their aftercare to provide patients with a cross-care structure. From our perspective, this approach increases the health awareness among patients and could have a sustainable impact. A short-term aerobic exercise could offer an innovative, cost-effective, and effective treatment approach in acute geriatric psychiatry with relevant benefits for patients, family members, and clinicians.

## Data Availability

The data that support the findings of this study are available upon request from the corresponding author. The data are not publicly available due to privacy or ethical restrictions. Study results are shared anonymously with participating physicians, referring physicians, patients, and the general medical community.

## Abbreviations

ADL: Activities of daily living
BDI II: Beck Depression Inventory
BDNF: Brain-derived neurotrophic factor
CAM: Confusion assessment method
CGI: Clinical global impression of change
CG: Control group
FED: Fluoxetin equivalents
GBD: Global Burden of Disease
HAMD: Hamilton Rating Scale for Depression
HR: Heart rate
ICD-10: International Statistical Classification of Diseases and Related Health Problems
IG: Intervention group
MMSE: Mini-Mental Status Examination
RCT: Randomized controlled trial
TAU: Treatment as usual
TUG: Timed Up and Go Test
RPE: Rate the perceived exertion
